# Secondary Metabolites of *Bacillus zhangzhouensis* from *Zygophyllum oxianum* and Their Antifungal and Plant Growth-Regulating Properties

**DOI:** 10.3390/plants14132058

**Published:** 2025-07-04

**Authors:** Zokir O. Toshmatov, Fazliddin A. Melikuziev, Ilkham S. Aytenov, Ma’ruf Z. Isokulov, Gulnaz Kahar, Tohir A. Bozorov, Daoyuan Zhang

**Affiliations:** 1 Key Laboratory of Desert and Oasis Ecology, Key Laboratory of Ecological Safety and Sustainable Development in Arid Lands, Xinjiang Institute of Ecology and Geography, Chinese Academy of Sciences, Urumqi 830011, China; zokirtoshmatov06@gmail.com (Z.O.T.); fmelikuziev23@gmail.com (F.A.M.); ilhamaytenov@gmail.com (I.S.A.); isoqulovmarufbek@gmail.com (M.Z.I.); gulinazikahaer20@mails.ucas.ac.cn (G.K.); 2 Laboratory of Bioresources and Stress Biology, Institute of Genetics and Plants Experimental Biology, Uzbek Academy of Sciences, Yukori-Yuz, Kibray 111226, Tashkent Region, Uzbekistan

**Keywords:** antagonistic bacteria, antibiotic, plant growth regulators, rhizosphere bacteria, medicinal plant

## Abstract

Plant species harbor diverse rhizospheric bacteria within their resilient root zones, serving as a valuable reservoir of bioactive microorganisms with strong potential for natural antifungal and plant growth-promoting applications. This study aimed to investigate the antagonistic potential of *Bacillus zhangzhouensis*, isolated from *Zygophyllum oxianum* in the Aral Sea region, Uzbekistan, against the fungal pathogen *Cytospora mali*. Due to its strong antifungal activity, *B. zhangzhouensis* was selected for bioactive compound profiling. Methanolic extracts were fractionated via silica and Sephadex gel chromatography, followed by antifungal screening using the agar diffusion method. A highly active fraction (dichloromethane/methanol, 9:1) underwent further purification, yielding twelve antifungal sub-fractions. Mass spectrometry analysis across positive and negative ion modes identified 2475 metabolites, with polar solvents—particularly methanol—enhancing compound recovery. Refinement using *Bacillus*-specific references identified six known antibiotics. Four pure compounds were isolated and structurally characterized using NMR: oleanolic acid, ursolic acid, cyclo-(Pro-Ser), and uracil. Their growth regulatory activity was assessed on *Amaranthus retroflexus*, *Nicotiana benthamiana*, triticale, and *Triticum aestivum* at concentrations of 5, 20, 100, and 500 mg L−1. All compounds negatively affected root growth in a concentration-dependent manner, especially in monocots. Interestingly, some treatments enhanced stem growth, particularly in *N. benthamiana*. These findings indicate that *B. zhangzhouensis* produces diverse bioactive compounds with dual antifungal and plant growth-modulatory effects, highlighting its potential as a biocontrol agent and a source of natural bioactive compounds.

## 1. Introduction

The genus *Zygophyllum* Boiss., belonging to the family Zygophyllaceae, comprises 39 species widely distributed across the flora of Central Asia, with 13 species endemic to Uzbekistan. These plants thrive in a range of harsh environmental conditions, including sandy and loamy soils, saline stony areas, and along irrigation channels and roadsides—particularly in the lower and middle regions of Uzbekistan [1]. *Zygophyllum* species are typically found in arid and semi-arid regions across Africa, the Mediterranean Basin, Central Asia, Australia, and the Americas and are recognized for their salt tolerance [2,3]. Traditionally, they have been extensively used in folk medicine throughout Arab and Asian countries [2,4], largely due to their content of biologically active compounds, particularly triterpene glycosides [5,6]. Among them, *Z. oxianum* is a desert-adapted shrub endemic to the Aral Sea region of Uzbekistan [1].

The rhizosphere, a zone surrounding plant roots, is recognized as one of the most complex ecosystems on Earth [7]. It harbors a vast array of microorganisms, with rhizospheric bacteria—particularly plant growth-promoting rhizobacteria (PGPR)—playing key roles in plant development and disease resistance. PGPR can directly enhance plant growth by producing phytohormones, solubilizing nutrients, and fixing nitrogen [8]. Indirectly, they contribute to plant health by suppressing soil-borne pathogens through competitive exclusion and antagonism [9]. Conversely, pathogenic bacteria can also colonize the rhizosphere by attempting to breach microbial defenses and bypass the plant’s immunity mechanisms [9]. It is well established that both plant genotype and soil type are major factors influencing the composition and structure of the rhizosphere microbiome [9,10]. Antagonistic bacteria, commonly found in the rhizosphere and phyllosphere, contribute to plant health and ecosystem stability by inhibiting harmful phytopathogens. They employ multiple mechanisms, such as competing for nutrients and niches, producing antimicrobial compounds, and releasing enzymes that degrade pathogen cell walls [11,12,13]. Beyond protection, many of these beneficial microbes also promote plant growth by enhancing nutrient uptake, synthesizing phytohormones, and improving soil quality [14].

Bacterial metabolites often include a variety of biologically active substances with antibacterial and antifungal properties [15,16]. Among these, *Bacillus* species are prominent producers of secondary metabolites and are considered a promising genus for agricultural applications [17,18]. The rapid mutation rates and resistance development among plant pathogens pose a serious threat to crop production, leading to substantial agricultural losses [19,20]. The application of naturally derived antimicrobial compounds produced by microorganisms is becoming increasingly essential in combating pathogenic microbes, particularly in the context of rising antimicrobial resistance. Notably, PGPR such as *Bacillus*, *Enterobacter*, and *Pseudomonas* have been isolated from members of the Zygophyllaceae family, including *Z. simplex*, in the Al Taif region, Saudi Arabia. These strains demonstrated antagonistic effects against plant pathogens such as *Fusarium oxysporum* and *Sclerotinia sclerotiorum* [20].

This study aimed to explore the bioactive compounds produced by *B. zhangzhouensis*, with a focus on its in vitro antagonistic activity, plant growth-promoting traits, and classification. During the investigation of secondary metabolites associated with rhizospheric bacteria isolated from the roots of *Z. oxianum*, a strain of *B. zhangzhouensis* was identified [21] and selected for further analysis. This strain was subjected to secondary metabolite extraction and screening. A combination of analytical techniques was employed to assess the antifungal properties of the extracted fractions. Furthermore, a chemical analysis of the methanolic extract from the bacterial culture led to the isolation of four compounds that are currently undergoing further characterization to evaluate their potential roles in promoting plant growth.

## 2. Material and Methods

### 2.1. Pathogen and Antagonism Screening

In the previous research [21], *B. zhangzhouensis* was isolated from *Z. oxianum* following the described methodology [21,22], and its antifungal activity against several plant pathogenic fungi was determined. To determine the antagonistic ability against *C. mali* obtained from previous work [23], a mixed medium consisting of Nutrient Agar (NA) and Potato Dextrose Agar (PDA) was used. A 1 cm diameter agar plug containing actively growing *C. mali* mycelium was excised from a PDA plate and placed at the center of the agar plate containing mixed medium. Bacterial isolates were then streaked at a distance of 2 cm from the fungal plug. Antagonistic activity was evaluated by measuring the inhibition of fungal mycelial growth, using the method described by Alenezi et al. [24]. The inhibition rate (I%) was calculated using the following formula:

I (%) = (1 − a/b) × 100

where “a” is the distance from the center of the fungal colony to the edge of fungal growth on the side facing the bacterial colony, and b is the radius of the fungal colony in the control plate (without bacteria).

### 2.2. Molecular Phylogeny

The 16S rRNA gene sequence of *B. zhangzhouensis* strain IGPEB-AS-01 (GenBank accession number: PP267998) [21] was compared against publicly available bacterial sequences in the GenBank database using the BLASTN algorithm. Multiple sequence alignments were conducted using CLUSTALW in MEGA11, and a phylogenetic tree was constructed using the maximum likelihood (ML) method based on the Tajima–Nei model. The robustness of the tree topology was evaluated with 500 bootstrap replicates [25,26,27].

### 2.3. Separation and Purification of Metabolites

A total of 20 L of active fermentation broth (LB broth: tryptone 10 g L^−1^, NaCl 10 g L^−1^, yeast extract 5 g L^−1^, pH 7.2) was obtained from *B. zhangzhouensis* strain IGPEB-AS-01, following an established protocol [22,28]. After centrifugation, the supernatant was carefully collected, and the fermentation products were dried. The resulting dried mass was extracted with methanol, and the extract was then concentrated using a DLAB RE100 rotary evaporator (DLAB, Beijing, China), yielding a concentrated crude extract.

For the separation of metabolites, silica gel powder with a mesh size of 200–300 (#80002, Qingdao Marine Chemical Company, Qingdao, China) was mixed with the crude extract and loaded onto a chromatography column (80 cm length, 5 cm diameter) containing 280 g of silica gel. The sample was extracted, mixed with silica gel, and processed into a fine powder using a DLAB RE100 rotary evaporator (Beijing, China). This powder was then loaded into the chromatography column. The column was first eluted with pure dichloromethane, and equal volumes of the effluent were collected. A gradient of dichloromethane and methanol (100:1, 18:1, 9:1, 4:1, and 1:1) was used to progressively flush out the remaining active components from the column, followed by a final cleanup with pure methanol. The collected fractions were concentrated and analyzed using thin-layer chromatography (TLC) to assess their antifungal activity, demonstrating the effectiveness of the separation process. Active fractions were further evaluated for antimicrobial activity using the agar diffusion method.

Gel chromatography was performed using Sephadex LH-20 (#GE17-0090-01, Cytiva, Marlborough, MA, USA), employing dichloromethane and stepwise methanol gradients for the effective separation of compounds. Sephadex LH-20 was swollen overnight in a 1:1 dichloromethane–methanol mixture before being packed into a 109 × 1.27 cm glass column. Elution fractions (5 mL) were collected in glass vials and monitored by TLC. The antifungal activity of the fractions was assessed using the agar diffusion method.

### 2.4. Thin-Layer Chromatography (TLC)

TLC was performed using 60 F254 silica gel on aluminum plates (Merck, Darmstadt, Germany). Spots were visualized under a UV lamp (λ_max_ = 254–365 nm), with additional detection using iodine vapors or vanillin–sulfuric acid, followed by heating at 105 °C for charring. During column chromatography, 100 mL fractions were collected using pure, analytical-grade solvents. The eluted solvents were then evaporated under reduced pressure at temperatures not exceeding 50 °C. For TLC analysis, 10 µg/µL of each compound was spotted onto the plates using fine capillary tubes, and the spots were detected in a UV chamber. The mobile phases employed for effective separation included hexane-ethyl acetate (4:1, 2:1) and dichloromethane–methanol (12:1, 9:1).

### 2.5. Antagonistic Activity Evaluation

To assess the antifungal activity of the chromatography fractions, the method by Bozorov et al. [22] was followed. Briefly, 3-mm diameter wells were punched into PDA plates using a hole puncher. Each fraction from the column chromatography was then carefully loaded into the wells under sterile conditions in a laminar flow cabinet. A 5 mm piece of fungal mycelium, grown on PDA, was placed in the center of each plate. The plates were incubated at 25 °C for 7 days, and fungal growth was monitored daily.

### 2.6. HPLC, Mass Spectrometry and NMR Analysis

High-performance liquid chromatography (HPLC) analysis was conducted using a Hitachi Chromaster HPLC system, which included a 1110 pump, a DT-230 column oven, a 1430 diode array detector, and a YMC C18 column (250 × 4.6 mm, 5 µm) (Hitachi, Tokyo, Japan). The analysis was carried out with EZChrom Elite software (A.04.07), and the mobile phases used were water, acetonitrile, and methanol.

The bacterial metabolite analysis of the studied extracts was performed using an liquid chromatography/mass spectrometry (LC/MS) system for metabolomics. This system comprised a Waters Acquity I-Class PLUS ultra-high-performance liquid chromatograph coupled with a Waters Xevo G2-XS QT high-resolution mass spectrometer, utilizing a Waters Acquity UPLC HSS T3 column (1.8 µm, 2.1 × 100 mm) at Biomarker Technologies (Beijing, China). For both negative and positive ion modes, phase A consisted of a 0.1% formic acid aqueous solution, and phase B was 0.1% formic acid in acetonitrile. The injection volume was 2 µL. During each data acquisition cycle, dual-channel data were acquired at both low and high collision energies simultaneously. The low collision energy was set to “off,” and the high collision energy range was 10–40 V, with a scanning frequency of 0.2 s per mass spectrum.

The parameters for the electrospray ionization (ESI) source were as follows: a capillary voltage of 2500 V (positive mode) or −2000 V (negative mode); a cone voltage of 30 V; an ion source temperature of 100 °C; a desolvation gas temperature of 500 °C; a backflush gas flow rate of 50 L/h; and a desolvation gas flow rate of 800 L/h. Raw data collected with MassLynx V4.2 were processed using Progenesis QI software (V.3.0) for peak extraction, alignment, and other data processing tasks. Compound identification was based on the METLIN online database and a self-constructed library. After normalizing the original peak area to the total peak area, identified compounds were further analyzed for classification and pathway information in the KEGG, HMDB, and LipidMaps databases.

For further structural elucidation, extensive spectroscopic data were collected, including ^1^H and ^13^C NMR spectra, using a JEOL AL300 FT-NMR spectrometer (JEOL Ltd., Tokyo, Japan) operating at 300 MHz and 75 MHz, respectively. Chemical shifts were reported in parts per million (ppm) relative to tetramethylsilane (TMS) as the internal reference (δ = 0.0 ppm), with coupling constants (J) provided in hertz (Hz). The analyses were conducted using CD3OD and DMSO-d_6_ as solvents.

### 2.7. Plant Growth Regulatory Activity of Isolated Compounds

The plant growth regulatory activity of the extracted compounds was evaluated using two dicotyledon species, *A. retroflexus* and *N. benthamiana*, and two monocotyledon species, triticale and *T. aestivum* following previous protocol [29]. To ensure accurate results, the seeds were surface sterilized using 75% ethanol. For the assessment, the four isolated compounds were diluted in methanol to achieve concentrations of 5, 20, 100, and 500 µg mL^−1^. Small seeds (*A. retroflexus* and *N. benthamiana*) were placed in cylindrical weighing bottles (40 × 25 mm), while larger seeds (triticale and *T. aestivum*) were tested in wider dishes (approximately 25 × 25 mm or 2.5 cm diameter). Each concentration was dish-lined with sterile filter paper, into which 2 mL of the solution was introduced. After the complete evaporation of methanol, 2 mL of distilled water was added, followed by the placement of test seeds, with 10 seeds allocated per dish. To provide optimal growth conditions, the Petri dishes were sealed with parafilm and stored in the dark at 25 °C. After a growth period of 7 days, measurements of root and shoot lengths were taken. Each bioassay was repeated three times, with 30 seedlings measured per concentration.

### 2.8. Statistical Analysis

All experimental data represent the means of at least three independent replicates. Comparisons between data sets were performed using a one-way ANOVA followed by Fisher’s PLSD post hoc test. A *p*-value of <0.05 was considered statistically significant. All statistical analyses were conducted using StatView software packages (https://statview.software.informer.com/, SAS Institute Inc., Cary, NC, USA). Figures were generated using Adobe Illustrator CS3 Version 13.0.0.

## 3. Result

### 3.1. Identification of Antagonistic Bacteria

**Figure 1:**
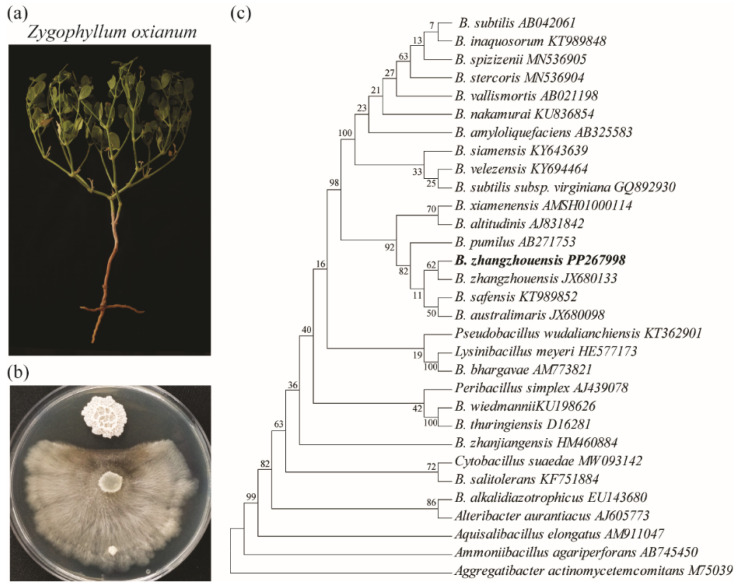
A phylogenetic analysis of the identified antagonistic bacteria. (**a**) *Z. oxianum* plant from Aral lake. (**b**) The co-cultivation of *B. zhangzhouensis* with *C. mali.* (**c**) The phylogenetic relationship of the antagonistic *B. zhangzhouensis* bacterium. The evolutionary history was inferred using the maximum likelihood (ML) method and the Tamura–Nei model. The percentage of trees in which the associated taxa clustered together is indicated next to the branches. Initial trees for the heuristic search were generated automatically by applying the Neighbor-Joining (NJ) and BioNJ algorithms to a matrix of pairwise distances estimated using the Tamura–Nei model. *A. elongatus*, *A. agariperforans*, and *Ag. actinomycetemcomitans* were used as outgroups.

To investigate the antagonistic effects on the pathogenic *C. mali*, a combination of microbiological, molecular, and analytical methods was employed. We utilized *C. mali*, a pathogenic fungus that causes cancer disease in economically important apple species, due to its rapid in vitro growth, which facilitates faster results in agar diffusion experiments compared to other fungal pathogens [21]. *Bacillus zhangzhouensis* IGPEB-AS-01, isolated from *Z. oxianum* growing in the Aral Sea region (Figure 1a), demonstrated strong antifungal activity against *C. mali* (Figure 1b). A online BLAST analysis of the 16S rRNA gene sequence revealed a 99.86% identity with *B. zhangzhouensis* (PP267998), confirming that the isolate belongs to this species (Figure 1c) [21].

### 3.2. Mass Spectrometry Analysis of Active Fractions

**Figure 2:**
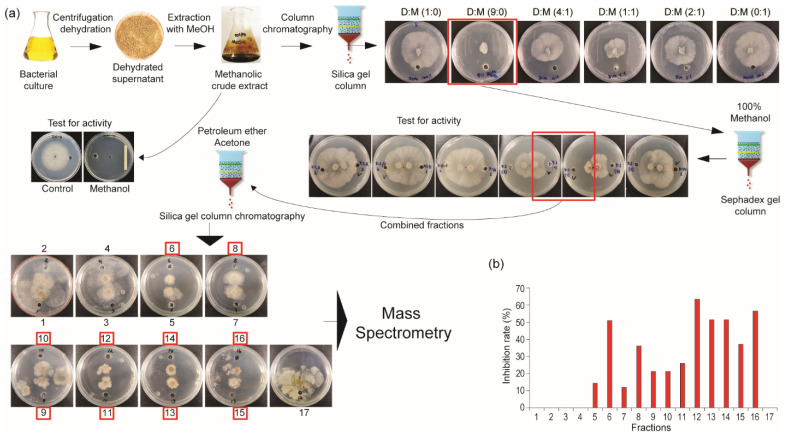
Bioactive antifungal compound isolation from bacterial extract through successive chromatographic steps and mass spectrometry. (**a**) The step-by-step workflow for the isolation of active fractions exhibiting antifungal activity. (**b**) Inhibition rates of the isolated active fractions.

To better understand the antifungal activity of this bacterium, a crude methanolic extract was subjected to chromatographic fractionation, including separations on silica gel and Sephadex columns. Fraction collection was guided by antifungal activity using the agar diffusion method. The further purification of the active fractions was carried out using high-performance liquid chromatography (HPLC) and mass spectrometry to identify the enriched antifungal compounds (Figure 2a). All fractions were individually tested for antifungal activity against *C. mali* in vitro. Among the seventeen fractions obtained, twelve showed varying levels of antifungal activity (Figure 2b). These active fractions were then analyzed by mass spectrometry to characterize their bioactive components.

**Figure 3:**
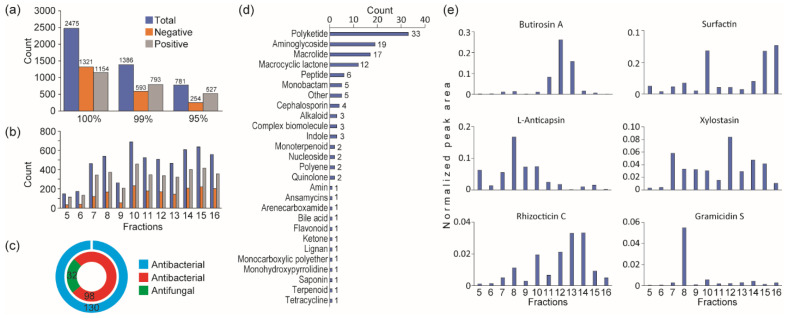
Mass spectrometry analysis of active fractions. (**a**) Statistics of metabolites identified from various ionization modes and number of metabolites at 100%, 99%, and 95% confidence levels. (**b**) Distribution of metabolites at 95% confidence across fractions exhibiting antifungal activity. (**c**) Reference-based functional descriptions of antibiotic metabolites obtained through database annotations. (**d**) Distribution of proposed antibiotics categorized by class. (**e**) Levels of potential antifungal compounds specific to *Bacillus* species across the fractions.

To characterize their chemical composition, twelve fractions exhibiting antifungal activity were analyzed by LC-ESI-QTOF-MS/MS under both positive and negative ionization modes. Metabolite identification was performed using the LC-QTOF platform, integrating both qualitative and quantitative metabolomics approaches across all twelve active fractions harboring antifungal activity. Mass spectrometry analysis detected a total of 13,623 peaks, from which 2475 metabolites were successfully annotated—1321 in negative mode and 1154 in positive mode (Figure 3a). The chemical profiles varied significantly depending on the extraction solvent used (Figure 3b). Notably, compounds such as alkaloids, steroids, lipids, and flavonoids were more abundant in extracts obtained using polar solvents, especially methanol.

Metabolite extraction and analysis at a 95% confidence level resulted in the identification of 781 metabolites, as summarized in Table S1. Among these, 32 metabolites were identified as antifungal and 98 as antibacterial (Figure 3c; Supplementary Material Table S1). These antibiotic candidates were categorized into 27 distinct groups (Figure 3d). To enhance prediction accuracy, only antibiotic compounds known to be derived from *Bacillus* species were considered. This refined analysis identified six antibiotics (gramicidin, butirosin, 2-deoxystreptamine, anticapsin, rhizocticins, and surfactin) specific to *Bacillus*, based on reference data [30,31,32,33,34,35] (Figure 3e). The distribution of these compounds varied across the different fractions.

### 3.3. Determination of Individual Compounds

**Figure 4:**
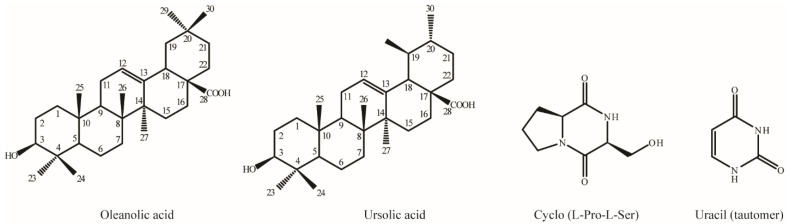
Chemical structures of four purified compounds isolated from *B. zhangzhouensis* extract.

Based on TLC analysis, non-active fractions 3 and 4 each contained a single compound, while the 17th fraction contained two distinct compounds. The 17th fraction was subsequently re-purified using silica gel column chromatography with the same solvent system. HPLC analysis confirmed the purity of the isolated compounds. For structural elucidation, the purified compounds were further analyzed using NMR spectroscopy (Figure 4). NMR data were obtained for oleanolic acid (13 mg) [36], ursolic acid (4 mg) [37], cyclo-(Pro-Ser) (5 mg) [38], and uracil (30 mg) [39], analyzed (Supplementary Data S1), and validated by comparison with reference data.

### 3.4. Growth Regulatory Activity of the Isolated Compounds

**Figure 5:**
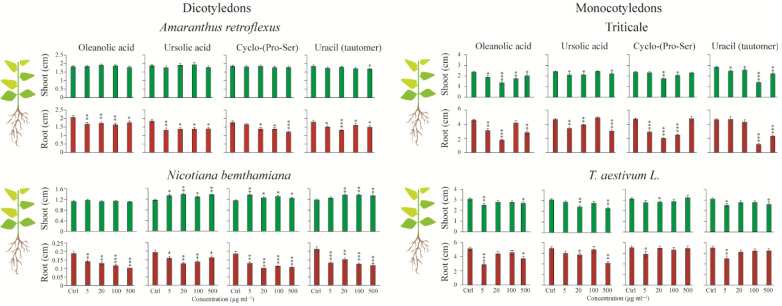
The effects of non-antibiotic compounds on monocotyledonous and dicotyledonous plants are evaluated. Data are presented relative to the water control. Mean (±SE) of ten replicates per line. Asterisks indicate significant differences (∗ *p* < 0.05; ∗∗ *p* < 0.01; ∗∗∗ *p* < 0.001) in Fisher’s PLSD test following an ANOVA.

Four concentrations (5, 20, 100, and 500 mg L^−1^) of each compound were tested on *N. benthamiana*, *A. retroflexus*, *T. aestivum* (wheat), and triticale. The effects were assessed by measuring seedling stem and root lengths. Overall, all tested compounds negatively inhibited root growth in a concentration-dependent manner. This inhibitory effect was more pronounced in dicotyledonous species, where root length was reduced up to 80% at higher concentrations (Figure 5). Monocotyledonous species also exhibited a concentration-dependent response, although sensitivity varied by species. For instance, both oleanolic and ursolic acids significantly inhibited root elongation in triticale and wheat at 5, 20, and 500 mg L^−1^, but not at 100 mg L^−1^. Notably, wheat showed no significant reduction in root length at 20 mg L^−1^ for oleanolic acid and at 5 mg L^−1^ for ursolic acid. Cyclo-(Pro-Ser) and uracil negatively affected root growth in *T. aestivum* only at 5 mg L^−1^, whereas in triticale, cyclo-(Pro-Ser) significantly reduced root length at 5, 20, and 100 mg L^−1^, and uracil at 100 and 500 mg L^−1^. In contrast, stem length did not exhibit a consistent dose-dependent trend. In *A. retroflexus*, stem length remained largely unaffected, except for a slight decrease caused by uracil at 500 mg L^−1^. Interestingly, ursolic acid, cyclo-(Pro-Ser), and uracil consistently promoted stem elongation in *N. benthamiana* across all concentrations. Meanwhile, monocotyledonous species (triticale and wheat) generally exhibited reduced stem length, though to varying degrees. Overall, triticale appeared more sensitive than wheat to all tested compounds in terms of both root and stem growth responses.

## 4. Discussion

This research highlights the biocontrol potential of *B. zhangzhouensis*, isolated from the rhizosphere of *Z. oxianum* in the Aral Sea region. Molecular identification verified *B. zhangzhouensis*, and its antifungal activity against *C. mali* emphasizes the value of investigating less-studied environments for discovering beneficial microbial resources. Previous research [22] established the potent antifungal activity of *B. zhangzhouensis* against various pathogenic fungi. The identification of *B. zhangzhouensis* as an antagonist of phytopathogenic fungi supports previous findings on the genus of *Bacillus*, known for producing a wide spectrum of antimicrobial metabolites [21,22,28,40,41,42]. Bacteria associated with plants contribute to defense and enhance plant immunity [43,44,45].

Using an integrated chromatographic approach, antifungal fractions were effectively separated and analyzed. This particular finding emphasizes that methanol is the most effective solvent for maximizing compound recovery. The subsequent fractionation and metabolite profiling using LC-ESI-QTOF-MS/MS enabled the annotation of over 2400 metabolites, with notable variation across fractions. Antibiotics known to be produced by *Bacillus* species highlight the extensive bioactive potential of the fractions [30,31,32,33,34,35]. Based on a survey of references, *Bacillus*-specific antibiotics such as anticapsin, surfactin, xylostasin, rhizocticin, and gramicidin S have been reported to exhibit antifungal activity, with the exception of butirosin A [30,32,34,46,47]. The antifungal agents showed variable distribution across the fractions, indicating a potential cooperative effect against fungal pathogens. Fractions 11 to 16, which exhibited strong antifungal activity, were likely associated with high levels of surfactin, xylostasin, and rhizocticin. These compounds may serve as the primary antifungal agents produced by *B. zhangzhouensis*.

The structural elucidation of compounds such as oleanolic acid, ursolic acid, cyclo-(Pro-Ser), and uracil highlights the chemical diversity of *B. zhangzhouensis* metabolites. These compounds exhibited distinct effects on plant growth, particularly root elongation, which was generally inhibited in a concentration-dependent manner across tested species. Notably, ursolic acid, cyclo-(Pro-Ser), and uracil consistently promoted stem elongation in *N. benthamiana*, in agreement with previous findings. For example, uracil has been reported to enhance stem growth in tomato at higher concentrations (500 mg L^−1^) [48]. Ursolic acid, known for its role in alleviating salt stress in rice, exerts beneficial effects by modulating oxidative stress, enhancing antioxidant activity, and regulating ion balance via nitric oxide signaling [49]. Additionally, both ursolic and oleanolic acids are well-characterized secondary plant metabolites involved in plant defense mechanisms, including responses to water deficit and pathogen infection [50]. Uracil, on the other hand, has been reported to inhibit germination and growth in certain leguminous species [51]. Cyclo-(Pro-Ser) is a cyclic dipeptide with known antifungal activity. In rice, it improves seedling vigor and reduces seed rot by disrupting fungal cell membranes, performing better than fungicides such as carbendazim at lower concentrations [52]. While cyclo-(Pro-Ser) and uracil are commonly produced by various bacterial taxa, reports of microbial production of ursolic and oleanolic acids are scarce and typically limited to engineered strains. This is the first report demonstrating the natural production of ursolic and oleanolic acids by *B. zhangzhouensis* under culture conditions.

The monocotyledonous species triticale and wheat exhibited a specific concentration-dependent response. In particular, low and/or high concentrations of the compounds affected growth parameters, whereas intermediate concentrations often had no significant effect. The dose-dependent phytotoxicity is consistent with classic toxicological responses, where increased concentrations of bioactive compounds lead to a stronger inhibition of plant growth. The variation in sensitivity between the two likely reflects genetic and physiological differences, such as in detoxification capacity or hormone signaling. Notably, the non-linear responses at intermediate doses may suggest hormetic or threshold effects, particularly in compounds like oleanolic and ursolic acids. The dose–response relationship in hormesis is typically biphasic, often taking the form of an inverted U-shaped or J-shaped curve. At intermediate concentrations, such as 100 mg L^−1^, the stimulatory effects observed at lower concentrations may be counteracted by the onset of inhibitory or toxic effects. This concentration may represent a “balancing point,” where the positive effects are no longer sufficient to produce a statistically significant increase. Additionally, triticale appeared to be more sensitive to the tested compounds compared to wheat. Collectively, these findings highlight the multifunctional nature of *B. zhangzhouensis* metabolites, revealing both growth-regulatory and protective properties, and support their potential application in sustainable plant health management.

*B. zhangzhouensis*, isolated from the rhizosphere of the desert plant *Z. oxianum*, exhibits strong antifungal activity and produces a diverse array of bioactive metabolites under in vitro conditions. Notably, this study is the first to report its production of ursolic and oleanolic acids in culture.

## Data Availability

The 16S rRNA gene sequence of *B. zhangzhouensis* can be found under GenBank accession number PP267998.

## Supplementary Materials

The following supporting information can be downloaded at https://www.mdpi.com/article/10.3390/plants14132058/s1, Supplemental Table S1: Predicted Antibiotic Compounds and Their Relative Abundance; Supplementary Data S1: NMR data for the compounds.
