# Supplementary material for: Secondary Metabolites of Bacillus zhangzhouensis from Zygophyllum oxianum and Their Antifungal and Plant Growth-Regulating Properties
Source: Plants (Basel). 2025 Jul 4;14(13):2058. doi: 10.3390/plants14132058 (PMC12252115; doi:10.3390/plants14132058)
Supplement: Supplementary file 1 [file plants-14-02058-s001.zip › plants-3666979-supplementary.pdf]

Supplemental table S1. Predicted Antibiotic Compounds and Their Relative Abundance

	Normalized peak area											
**Metabolite**	**F5**	**F6**	**F7**	**F8**	**F9**	**F10**	**F11**	**F12**	**F13**	**F14**	**F15**	**F16**
(-)-Germacrene D	0.00	0.00	0.04	0.08	0.04	0.01	0.00	0.00	0.00	0.00	0.00	0.00
(7R)-7-(4-Carboxybutanamido)cephalosporanate	0.01	0.00	0.01	0.02	0.01	0.02	0.00	0.00	0.00	0.00	0.00	0.00
10-Deoxymethymycin	0.00	0.00	0.00	0.00	0.00	0.00	0.00	0.00	0.00	0.00	0.01	0.02
10-Deoxymethynolide	0.00	0.01	0.01	0.01	0.64	0.03	0.03	0.02	0.03	0.03	0.04	0.03
Xylostasin	0.00	0.00	0.06	0.03	0.03	0.03	0.02	0.08	0.03	0.05	0.04	0.01
L-Anticapsin	0.06	0.01	0.05	0.17	0.07	0.07	0.02	0.02	0.00	0.01	0.02	0.00
Butirosin A	0.00	0.00	0.01	0.01	0.00	0.01	0.08	0.26	0.16	0.02	0.01	0.00
Surfactin	0.02	0.01	0.02	0.03	0.01	0.14	0.02	0.02	0.01	0.04	0.14	0.15
Rhizocticin C	0.00	0.00	0.00	0.01	0.00	0.02	0.01	0.02	0.03	0.03	0.01	0.00
Gramicidin S	0.00	0.00	0.00	0.05	0.00	0.01	0.00	0.00	0.00	0.00	0.00	0.00
Neamine	0.01	0.02	0.01	0.02	0.01	0.02	0.01	0.01	0.01	0.01	0.01	0.00
tunicamycin	0.01	0.01	0.03	0.08	0.05	1.54	0.66	0.36	0.14	0.16	0.18	0.16
Aphidicolin	0.01	0.11	0.03	0.02	0.00	0.01	0.02	0.02	0.01	0.02	0.02	0.03
L-Arginine	0.00	0.00	0.00	0.00	0.00	0.04	0.00	0.00	0.00	0.00	0.00	0.00
Prodigiosin	0.00	0.01	0.02	0.01	0.00	0.02	0.18	0.27	0.18	0.09	0.04	0.03
AGELASINE	0.05	0.03	1.11	0.07	0.02	0.12	0.14	0.13	1.01	0.06	0.07	0.02
10-Oxo-11-octadecen-13-olide	0.00	0.60	0.06	0.02	0.00	0.01	0.01	0.00	0.01	0.02	0.01	0.01
13-Dihydrodaunorubicin	0.00	0.00	0.01	0.12	0.06	0.15	0.04	0.03	0.03	0.05	0.05	0.06
19-Chloroproansamitocin	0.00	0.00	0.00	0.00	0.00	0.00	0.00	0.00	0.00	0.00	0.00	0.02
1-Oxo-1H-2-benzopyran-3-carboxaldehyde	0.00	0.00	0.00	0.23	0.01	0.03	0.00	0.00	0.00	0.00	0.00	0.01
2'-Deamino-2'-hydroxyneamine	0.00	0.00	0.00	0.01	0.00	0.01	0.00	0.01	0.01	0.06	0.04	0.02
2'''-N-Acetyl-6'''-deamino-6'''-hydroxyparomomycin II	0.00	0.01	0.02	0.05	0.01	0.01	0.01	0.01	0.01	0.01	0.02	0.03
3-(3-Hydroxyphenyl)propanoic acid	0.00	0.00	0.00	0.13	0.05	0.03	0.01	0.00	0.00	0.01	0.01	0.00
3''-Deamino-3''-hydroxykanamycin B	0.00	0.00	0.01	0.36	0.00	0.02	0.01	0.01	0.01	0.02	0.02	0.01
3''-Oxogentamicin A2	0.00	0.00	0.01	0.04	0.00	0.02	0.03	0.03	0.03	0.05	0.08	0.23
3''-Oxoribostamycin	0.00	0.00	0.00	0.00	0.00	0.00	0.00	0.00	0.00	0.01	0.02	0.01
4-Hydroxyphenylglyoxylate	0.00	0.00	0.00	0.05	0.00	0.02	0.01	0.00	0.00	0.00	0.00	0.00
5a,11a-Dehydrooxytetracycline	0.00	0.00	0.00	0.01	0.01	0.02	0.01	0.00	0.00	0.00	0.00	0.00
5-Hydroxyferulate	0.00	0.00	0.00	0.03	0.01	0.01	0.00	0.00	0.00	0.00	0.00	0.00
6'''-Deamino-6'''-hydroxyneomycin C	0.00	0.00	0.01	0.00	0.00	0.00	0.04	0.01	0.00	0.00	0.00	0.00
6'''-Deamino-6'''-oxoneomycin C	0.00	0.00	0.00	0.08	0.01	0.02	0.01	0.01	0.01	0.03	0.03	0.01
6'-Oxogentamicin X2	0.00	0.00	0.00	0.10	0.05	0.04	0.01	0.01	0.01	0.02	0.03	0.02
6'-Oxokanamycin X	0.00	0.02	0.06	0.01	0.00	0.00	0.00	0.00	0.00	0.00	0.00	0.00
6-pentadecyl Salicylic Acid	0.01	0.00	0.00	0.01	0.00	0.01	0.00	0.00	0.01	0.01	0.01	0.02
7a-Hydroxy-O-carbamoyl-deacetylcephalosporin C	0.00	0.00	0.00	0.00	0.01	0.04	0.00	0.00	0.00	0.00	0.00	0.00
7-Geranylformononetin	0.00	0.00	0.00	0.00	0.00	0.01	0.03	0.42	0.53	0.30	0.10	0.11
8,8a-Deoxyoleandolide	0.00	0.01	0.12	0.01	0.00	0.02	0.01	0.01	0.00	0.01	0.01	0.00
Myristic acid	0.02	0.14	0.02	0.02	0.01	0.01	0.00	0.00	0.00	0.00	0.01	0.01

Brevianamide F	0.01	0.01	0.04	0.38	0.03	0.25	0.14	0.35	0.39	0.85	0.39	0.06
Anisomycin	0.00	0.00	0.01	0.06	0.04	0.27	0.33	0.23	0.13	0.31	1.51	0.46
Astringin	0.00	0.00	0.00	0.01	0.01	0.01	0.02	0.01	0.01	0.02	0.02	0.21
Aurachin B	0.01	0.01	0.02	0.01	0.00	0.01	0.00	0.01	0.00	0.00	0.01	0.01
Aurachin C	0.00	0.00	0.00	0.01	0.00	0.01	0.00	0.01	0.00	0.00	0.00	0.08
Avermectin A1a	0.00	0.01	0.03	0.04	0.01	0.04	0.02	0.02	0.03	0.02	0.02	0.02
Avermectin A1a aglycone	0.00	0.00	0.01	0.07	0.47	0.03	0.01	0.01	0.01	0.04	0.05	0.02
Avermectin A1a monosaccharide	0.00	0.00	0.01	0.01	0.00	0.01	0.04	0.01	0.01	0.01	0.01	0.01
Avermectin A2a aglycone	0.04	0.00	0.04	0.03	0.01	0.03	0.24	0.44	0.13	0.05	0.04	0.03
Avermectin A2a monosaccharide	0.02	0.02	0.02	0.01	0.00	0.00	0.00	0.00	0.00	0.00	0.00	0.00
Avermectin B1a aglycone	0.00	0.00	0.02	0.01	0.00	0.02	0.01	0.01	0.02	0.03	0.01	0.01
Avermectin B1b aglycone	0.02	0.02	0.02	0.04	0.01	0.04	0.44	0.54	0.09	0.04	0.03	0.02
Avermectin B1b monosaccharide	0.00	0.00	0.00	0.02	0.00	0.07	0.01	0.00	0.00	0.01	0.01	0.00
Avermectin B2a	0.00	0.00	0.02	0.02	0.01	0.03	0.01	0.01	0.01	0.02	0.02	0.02
Avermectin B2a monosaccharide	0.00	0.00	0.01	0.01	0.01	0.03	0.01	0.01	0.01	0.02	0.01	0.00
Avermectin B2b aglycone	0.00	0.00	0.01	0.37	0.02	0.05	0.01	0.01	0.02	0.02	0.02	0.01
Bisdemethoxycurcumin	0.02	0.41	0.09	0.06	0.04	0.05	0.02	0.01	0.00	0.00	0.00	0.00
Candicidin D	0.00	0.00	0.01	0.04	0.00	0.01	0.00	0.00	0.00	0.00	0.00	0.00
Cannabidiol	0.00	0.00	0.01	0.01	0.00	0.02	0.03	0.60	1.04	0.34	0.08	0.01
Cephalosporin C	0.00	0.00	0.00	0.01	0.00	0.38	0.14	0.01	0.00	0.00	0.00	0.04
Cinnamaldehyde	0.00	0.00	0.03	0.01	0.00	0.00	0.00	0.01	0.00	0.01	0.01	0.01
Coumarin	0.00	0.00	0.00	0.03	0.01	0.02	0.00	0.00	0.00	0.00	0.00	0.00
Dicaffeoylputrescine	0.00	0.01	0.02	2.17	0.12	0.11	0.29	0.30	0.16	0.08	0.07	0.08
Dihydrocoumarin	0.00	0.00	0.00	0.02	0.01	0.01	0.00	0.00	0.00	0.00	0.00	0.00
Dihydroechinofuran	0.00	0.01	0.02	0.21	0.12	0.07	0.04	0.07	0.06	0.06	0.06	0.08
Dihydrokaempferol	0.02	0.00	0.01	0.00	0.03	0.00	0.00	0.00	0.00	0.00	0.00	0.00
Dioscin	0.11	0.00	0.03	0.12	0.03	0.07	0.13	0.02	0.08	0.13	0.03	0.03
Doxycycline	0.00	0.00	0.00	0.45	0.01	0.02	0.01	0.02	0.01	0.01	0.01	0.02
Epothilone A	0.11	0.02	0.03	0.08	0.07	0.29	0.06	0.01	0.00	0.00	0.00	0.00
Erythromycin D	0.00	0.00	0.00	0.01	0.03	0.00	0.00	0.00	0.00	0.00	0.00	0.00
Exalamide	0.00	0.00	0.01	0.03	0.01	0.06	0.08	0.27	0.18	0.08	0.04	0.01
Feruloylputrescine	0.00	0.00	0.89	0.13	0.00	0.08	0.01	0.01	0.01	0.02	0.02	0.03
fumagillin	0.00	0.00	0.02	0.11	0.03	0.07	0.03	0.02	0.02	0.03	0.02	0.02
Geldanamycin	0.00	0.00	0.01	0.32	0.01	0.05	0.01	0.01	0.01	0.02	0.02	0.01
Gentamicin A	0.00	0.01	0.01	0.03	0.01	0.04	0.05	0.12	0.07	0.11	0.15	0.12
Gentamicin C2	0.01	0.01	0.02	0.09	0.33	0.04	0.02	0.04	0.04	0.06	0.07	0.04
Ginkgolic Acid	0.01	0.00	0.02	0.02	0.02	0.02	0.19	0.06	0.07	0.03	0.04	0.01
globomycin	0.01	0.01	0.03	0.06	0.06	0.07	0.03	0.04	0.02	0.03	0.03	0.03
Immunomycin	0.01	0.03	0.04	0.02	0.01	0.03	0.01	0.01	0.06	0.05	0.03	0.03
Oxindole	0.01	0.00	0.01	0.03	0.01	0.03	0.20	0.21	0.34	0.42	0.24	0.20
indolmycin	0.01	0.00	0.01	0.07	0.01	0.02	0.01	0.01	0.01	0.01	0.01	0.01

Isonocardicin A	0.00	0.00	0.00	0.01	0.00	0.09	0.04	0.03	0.01	0.00	0.00	0.00
Kanamycin B	0.00	0.00	0.01	0.03	0.02	0.03	0.01	0.01	0.01	0.02	0.01	0.01
Kanamycin X	0.00	0.00	0.01	0.01	0.00	0.01	0.01	0.04	0.11	0.03	0.03	0.02
L-4-Hydroxyphenylglycine	0.00	0.00	0.00	0.00	0.00	0.00	0.00	0.00	0.00	0.01	0.03	0.01
Lariciresinol	0.00	0.01	0.02	0.05	0.01	0.04	0.58	0.04	0.02	0.03	0.06	0.03
Lithocholic acid	0.00	0.00	0.01	0.00	0.00	0.01	0.09	0.03	0.01	0.01	0.01	0.09
L-Oleandrosyl-oleandolide	0.00	0.00	0.01	0.18	0.01	0.03	0.01	0.01	0.01	0.02	0.03	0.01
L-Olivosyl-oleandolide	0.00	0.00	0.00	0.05	0.01	0.02	0.01	0.01	0.01	0.01	0.01	0.00
Malvidin 3-O-glucoside	0.00	0.00	0.00	0.04	0.00	0.00	0.00	0.00	0.00	0.00	0.00	0.00
Megalomicin B	0.01	0.11	0.12	0.10	0.03	0.16	0.08	0.06	0.05	0.10	0.17	0.16
Megalomicin C1	0.02	0.02	0.13	0.28	0.07	0.16	0.06	0.04	0.06	0.16	0.15	0.18
Megalomicin C2	0.00	0.00	0.01	0.01	0.00	0.01	0.00	0.00	0.01	0.01	0.02	0.03
Methyl cinnamate	0.00	0.00	0.00	0.00	0.00	0.00	0.00	0.00	0.00	0.00	0.00	0.02
Methymycin	0.01	0.00	0.02	0.01	0.00	0.16	0.07	0.16	0.26	0.03	0.02	0.01
Mithramycin	0.00	0.00	0.00	0.14	0.00	0.00	0.00	0.00	0.00	0.00	0.00	0.01
coformycin	0.01	0.01	0.02	0.17	0.05	0.25	0.30	0.07	0.07	0.11	0.44	0.10
Myxalamid A	0.10	0.06	0.23	0.40	0.03	0.23	0.73	0.30	0.25	0.10	0.20	0.04
Myxalamid S	0.01	0.01	0.05	0.23	0.02	0.11	0.25	0.30	0.16	0.10	0.12	0.03
myxothiazol	0.00	0.00	0.00	0.02	0.01	0.05	0.05	0.02	0.01	0.02	0.02	0.02
N-(p-Hydroxyphenyl)ethyl p-hydroxycinnamide	0.00	0.00	0.01	0.02	0.01	0.05	0.04	0.03	0.03	0.04	0.24	0.02
N-Acetyltyramine	0.02	2.70	0.06	0.00	0.00	0.00	0.00	0.00	0.04	0.04	0.06	0.14
Natamycin	0.00	0.00	0.00	0.02	0.00	0.00	0.00	0.00	0.00	0.00	0.00	0.00
Nebramycin 5'	0.00	0.00	0.01	0.01	0.00	0.11	0.02	0.01	0.01	0.01	0.01	0.01
Nebramycin factor 4	0.00	0.00	0.01	0.13	0.01	0.02	0.02	0.01	0.00	0.00	0.00	0.00
Neomethymycin	0.10	0.06	0.20	0.16	0.05	0.17	0.07	0.04	0.04	0.06	0.01	0.02
Neopikromycin	0.09	0.03	0.13	0.17	0.01	0.78	3.30	1.43	0.44	0.25	0.60	0.12
Nystatin A1	0.00	0.02	0.01	0.00	0.00	0.02	0.00	0.00	0.00	0.01	0.01	0.00
Oleandomycin	0.00	0.00	0.00	0.00	0.00	0.03	0.00	0.00	0.00	0.00	0.00	0.00
Paromomycin	0.00	0.01	0.01	0.03	0.01	0.03	0.11	0.09	0.02	0.02	0.01	0.01
p-CHLOROPHENYLALANINE	0.02	0.01	0.03	0.05	0.02	0.06	0.01	0.01	0.01	0.01	0.01	0.02
Pikromycin	0.00	0.00	0.01	0.07	0.58	0.05	0.02	0.03	0.04	0.05	0.10	0.00
Piplartine	0.00	0.00	0.01	0.02	0.00	0.49	0.65	0.27	0.03	0.02	0.07	0.03
Poinsettifolin B	0.00	0.00	0.01	0.04	0.01	0.05	0.05	1.18	1.46	0.63	0.25	0.16
Proansamycin X	0.00	0.00	0.00	0.04	0.00	0.01	0.00	0.00	0.00	0.00	0.00	0.00
Procaterol	0.01	0.02	0.03	0.52	0.05	0.15	0.07	0.07	0.11	0.14	0.10	0.10
Protorifamycin I	0.00	0.00	0.01	0.05	0.00	0.01	0.00	0.00	0.00	0.00	0.00	0.00
Ribostamycin	0.01	0.01	0.14	0.03	0.01	0.03	0.02	0.10	0.02	0.03	0.02	0.01
Rifamycin	0.00	0.00	0.00	0.03	0.00	0.00	0.00	0.00	0.00	0.00	0.00	0.00
Rifamycin B	0.00	0.00	0.00	0.14	0.00	0.01	0.00	0.01	0.01	0.01	0.01	0.01
Roseoflavin	0.00	0.00	0.00	0.01	0.01	0.04	0.00	0.00	0.01	0.01	0.00	0.00
Rotenone	0.01	0.00	0.02	0.02	0.01	0.25	0.34	0.07	0.00	0.00	0.02	0.00

Salbostatin	0.05	0.02	0.09	0.00	0.00	0.02	0.01	0.01	0.00	0.00	0.00	0.00
Salinomycin	0.11	0.52	0.75	0.76	0.29	0.46	0.37	0.35	0.29	0.36	0.32	0.36
sinefungin	0.00	0.00	0.01	0.58	0.17	0.03	0.01	0.01	0.00	0.01	0.01	0.00
Sirolimus	0.01	0.01	0.02	0.03	0.01	0.04	0.09	0.07	0.01	0.03	0.03	0.03
Solasodine	0.00	0.00	0.01	0.01	0.01	0.02	0.10	0.04	0.02	0.09	0.14	0.03
Tabtoxinine-delta-lactam	0.00	0.00	0.00	0.00	0.00	0.00	0.00	0.00	0.00	0.00	0.00	0.02
Tetracycline	0.00	0.00	0.01	0.59	0.01	0.04	0.02	0.02	0.02	0.02	0.02	0.02
trans-Isohumulone	0.05	0.07	0.01	0.04	0.01	0.01	0.01	0.00	0.00	0.00	0.00	0.01
Validamycin B	0.00	0.00	0.00	0.02	0.03	0.02	0.01	0.01	0.01	0.02	0.02	0.01
Vulgarone A	0.00	0.00	0.10	0.01	0.00	0.01	0.00	0.00	0.00	0.01	0.03	0.00

Supplementary data S1. NMR data for the compounds.

NMR data for oleanolic acid. ^{1}H NMR (600 MHz, MeOD) δ 5.24 (1H, t, *J* = 3.7 Hz, H-12), 3.15 (1H, dd, *J* = 11.4, 4.6 Hz, H-3), 2.85 (1H, dd, *J* = 13.8, 4.2 Hz, H-18), 2.01 (1H, td, *J* = 13.6, 4.1 Hz, H-16), 1.94 – 1.86 (2H, m, H-11), 1.81 – 1.72 (2H, m, H-7, 15), 1.69 (1H, t, *J* = 13.7 Hz, H-19), 1.66 – 1.60 (2H, m, H-1, 2), 1.60 – 1.57 (2H, m, H-9, 16), 1.57 – 1.52 (2H, m, H-6, 2), 1.50 (1H, dd, *J* = 12.6, 4.0 Hz, H-22), 1.46 – 1.40 (1H, m, H-6), 1.38 (1H, dd, *J* = 13.9, 4.1 Hz, H-21), 1.32 (1H, dt, *J* = 12.5, 3.1 Hz, H-22), 1.16 (3H, s, H-27), 1.15 – 1.10 (1H, m, H-19), 1.08 (1H, dt, *J* = 13.8, 3.5 Hz, H-15), 0.97 (3H s, H-23), 0.94 (6H, d, *J* = 2.2 Hz, H-25, 30), 0.91 (3H, s, H-29), 0.82 (3H, s, H-26), 0.78 (3H, s, H-24), 0.76 (1H, dd, *J* = 11.8, 2.0 Hz, H-5). ^{13}C NMR (151 MHz, MeOD) δ 181.91 (C-28), 145.23 (13), 123.64 (12), 79.72 (C-3), 56.76 (C-5), 49.57 (C-9), 47.65 (C-17), 47.26 (C-19), 42.89 (C-14), 42.75 (C-18), 40.56 (C-18), 39.84 (C-1), 38.17 (C-8), 34.90 (C-21), 34.02 (C-22), 33.83 (C-7), 33.57 (C-29), 31.62 (C-20), 28.84 (C-23), 28.74 (C-23), 27.87 (C-2), 26.39 (C-27), 24.52 (C-11), 24.06 (C-30), 23.98 (C-16), 19.50 (C-6), 17.72 (C-26), 16.31 (C-24), 15.89 (C-25)

NMR data for ursolic acid. ^{1}H NMR (600 MHz, MeOD) δ 5.23 (1H, t, J = 3.8 Hz, H-12), 3.15 (1H, dd, J = 11.6, 4.6 Hz, H-3), 2.20 (1H, d, J = 13.4 Hz, H-18), 2.04 (1H, td, J = 13.4, 4.3 Hz, H-16), 1.94 (2H, dd, J = 8.6, 4.2 Hz, H-11), 1.66 – 1.62 (1H, m, H-33), 1.56 (1H, dt, J = 13.3, 4.5 Hz, H-6), 1.47 – 1.29 (2H, m, H-20, 6), 1.12 (3H, s, H-27), 0.98 (3H, s, H-23), 0.97 (3H, s, H-29), 0.96 (3H, s, H-25), 0.89 (3H, d, J = 6.5 Hz, H-30), 0.85 (3H, s, H-26), 0.78 (3H, s, H-24), 0.77 – 0.74 (1H, m, H-5). ^{13}C NMR (151 MHz, MeOD) δ 181.70 (C-28), 139.66 (C-13), 126.89 (C-12), 79.70 (C-3), 56.75 (C-5), 54.38 (C-18), 49.85 (C-17), 49.57 (C-9), 43.25 (C-14), 40.78 (C-8), 40.43 (C-19), 40.42 (C-20), 40.00 (C-1), 39.84 (C-4), 38.12 (C-22), 38.11 (C-10), 34.34 (C-7), 31.78 (C-21), 29.22 (C-15), 28.77 (C-23), 27.90 (C-2), 25.33 (C-16), 24.36 (C-11), 24.09 (C-27), 21.57 (C-29), 19.48 (C-6), 17.81 (C-26), 17.65 (30), 16.38 (C-24), 16.02 (C-25)

NMR data for Cyclo-(Pro-Ser). White solid. ^{1}H NMR (600 MHz, CD_3OD, δ, ppm, J/Hz): 4.12, dd (10.7, 6.5 H-6) 3.66, q (3.4 H-9) 3.52, ddd (11.0, 5.4, 3.2 H-10), 3.33, (1H m H-3a), 3.44, dt (11.7, 8.4 H-3b), 1.70, (1H, m, H-5a) 2.14, (1H, m, H-5b), 1.86 (2H, m, H-4). ^{13}C NMR (150 MHz, CD_3OD, δ, ppm): 164.64 (C-1), 169.05 (C-7), 63.57 (C-10), 58.17 (C-6), 59.63(C-9), 44.82 (C-3), 28.77 (C-5), 21.52 (C-4)

NMR data for Uracil. White powder solid. 1H-NMR (DMSO-d6): 10.880 (1H, s), 10.709 (1H, s), 7.235 (1H, d), 5.297 (1H, d). 13C-NMR (DMSO-d6): 164.8 (C-4), 152.0 (C-2), 142.7 (C-2), 100.7 (C-6). Compared with 1HNMR and 13C-NMR, spectral data with those reported in the literature its structure was confirmed as uracil.
